# First record of *Diplotaxis kohlaanensis* (Brassicaceae) for the eastern African flora with taxonomical and phytogeographical remarks

**DOI:** 10.3897/BDJ.13.e161978

**Published:** 2025-10-07

**Authors:** Salvatore Cambria, Manuela Porrovecchio, Adriana Santanello, Pietro Minissale, Gian Pietro Giusso del Galdo

**Affiliations:** 1 Department of Biological, Geological and Environmental Science, University of Catania, Via A. Longo 19, I - 95125, Catania, Italy Department of Biological, Geological and Environmental Science, University of Catania, Via A. Longo 19, I - 95125 Catania Italy

**Keywords:** biogeography, chasmophilous vegetation, Ethiopia, morphology, taxonomy

## Abstract

**Background:**

*Diplotaxis* (Brassicaceae) is a genus widely distributed in the temperate areas of Europe, Asia and Africa, reaching its greatest diversity in NW Africa, Cape Verde and the Mediterranean area. In eastern Africa, this genus is only represented by *D. harra* (Forssk.) Boiss. in Somalia and Djibouti, while no native species of *Diplotaxis* have been recorded from Ethiopia.

**New information:**

The finding of *Diplotaxis kohlaanensis* A.G. Mill. & J.A. Nyberg, originally described from northern Yemen, is here reported for the first time in the Simien Mountains. Our finding has a particular phytogeographical and ecological significance, highlighting the relevant floristic relationships of the Arabian Peninsula mountains with the Ethiopian highlands. In addition, our investigations confirm the outstanding number of exclusive taxa shared by these two areas testifying to the past phytogeographical connection between these two territories. *D. kohlaanensis* is localised on the high-altitude cliffs, which represent a highly conservative environment for relict species. Finally, the taxonomical relationships of this taxon within the *Diplotaxis harra* complex are also examined.

## Introduction

*Diplotaxis* DC., a genus of Brassicaceae Burn. (tribe *Brassiceae* DC.), is mainly distributed in the temperate areas of Europe, Asia and Africa, including 30-40 species (Martinez-Laborde 1988, Warwick and Hall 2009, Pignone and Martínez-Laborde 2010, Gómez-Campo 2011, POWO 2025) with the greatest diversity in NW Africa, Cape Verde and the Mediterranean area (Martín and Sánchez-Yélamo 2000, Rustan 2008). According to the Flora of Ethiopia and Eritrea (Edwards et al. 2000), the most recent academic flora dealing with these countries, no species belonging to this genus occurs in Ethiopia, while other sources (Oliver 1868, Pignone and Martínez-Laborde 2010, POWO 2025) report the presence of weedy species *D. erucoides* L. For the remaining eastern African countries, the only known native species of *Diplotaxis* is *D. harra* (Forssk.) Boiss., occurring in northern Somalia and Djibouti (Thulin 1993). This species, originally placed by Schulz (1919) in the section Catocarpum, shows rather uncertain relationships and a quite isolated position within the genus *Diplotaxis* (Pradhan et al. 1992, Warwick et al. 1992, Martín and Sánchez-Yélamo (2000). Besides, this taxon is morphologically very variable (Martínez-Laborde 1991) and widely distributed from the (sub-)desertic territories of North Africa eastwards to Afghanistan and Pakistan (Zohary 1966, Pottier-Alapetite 1979, Fennane 1999, POWO 2025). Two closely-allied taxa, *D. crassifolia* (Raf.) DC. and *D. lagascana* DC., sometimes treated at subspecific level, are also known from Sicily (Italy) and southern Spain, respectively (Castroviejo et al. 1993, Pignatti et al. 2019, Brullo and Brullo 2020). Besides, the perennial populations of *D. harra* from North Africa, alternatively referred by various authors (Maire 1965, Pottier-Alapetite 1979, Fennane 1999) to the subsp. crassifolia and *lagascana*, but clearly distinguished by the morphology of the leaves and siliques, are provisionally attributed to the variability of the species, although further taxonomic, ecological and chorological investigations are desirable. The *D. harra* complex includes all the endemic taxa recorded from Cape Verde (Rustan 2008), whose origin is quite recent (Quaternary) likely from the western Saharan populations of *D. harra* as proposed by Franzke et al. (2017). Regarding the southern boundaries of *D. harra*, it reaches the central-southern part of the Arabian Peninsula and, particularly, Saudi Arabia, Yemen and Oman (Miller and Cope 1996), where Miller and Nyberg (1994) found and described a new closely-related species named *D. kohlaanensis* from the mountain area of Kuhlan (northern Yemen). According to these authors, this taxon is morphologically and ecologically quite distinct from *D. harra*, especially for its shrubby habit, corolla with longer petals and larger siliques. Whereas, relating to its ecology, it occurs on carbonatic or calcarenitic rocks located at high altitudes (2,300-3,000 m a.s.l.), featuring cool and humid environmental conditions where *D. harra* is typically found in the Arabian Peninsula. Recently, *D. kohlaanensis* was found by Brinkmann et al. (2009) also in the Jabal al Akhdar mountain range (Oman), although this record should be considered at least dubious as the ecological context seems unlikely for the species. However, other reports for Oman, such as those of the specimens in the GBIF database from the Dhofar Mountains, seem to demonstrate the presence of the species in that country.

During field surveys on the Afromontane and Afroalpine vegetation of the Simien Mts. (north-western Ethiopia), a population of an unknown species belonging to the genus *Diplotaxis* was found on the vertical cliffs just below the peak of Mt. Inatye, at an altitude between 3,700 and 3,900 m a.s.l. Further field and herbarium investigations allowed us to attribute this plant to the aforementioned *D. kohlaanensis* due to the peculiar morphological features of the stems, leaves, flowers and siliques. Our finding is quite relevant since it represents the first record of a native species of the genus *Diplotaxis* in Ethiopia, thus contributing to update the already significant pool of species shared by the Ethiopian highlands and the mountains of the southern Arabian Peninsula. This relatively surprising discovery in a territory that is quite well known from a floristic point of view, at least in the higher areas (Puff and Nemomissa 2001, Puff and Nemomissa 2005), is probably related to the localisation of the species on vertical walls that are difficult to access and perhaps also to its flowering period in the dry season. A detailed morphological description of the plants belonging to the surveyed population, as well as the phenology, conservation, distribution and ecology of the species in Ethiopia is discussed in this paper. Furthermore, a taxonomic review of the currently recognised taxa within the *D. harra* species complex is presented with the elaboration of a key. Finally, the floristic relationships between the Simien Mountains and the mountain ranges of the southern Arabian Peninsula are analysed in detail, highlighting the vegetation types that host the greatest number of shared species between eastern Africa and south-western Asia.

## Materials and methods

The study area is localised in the north-western part of Ethiopia and, in particular, in the Simien Mts. (13°140' N, 38°210' E) where the highest peak of Ethiopia is found (Mt. Ras Dejen, 4,540 m a.s.l.). This massif is the remnant of a major Oligo-Miocene shield volcano, deeply eroded by the Tekeze River and its tributaries, surrounding the massif (Kieffer et al. 2004). A peculiar feature is the presence of a steep slope of ca. 1,000 m connecting the high plateau to the lowlands.

The areas exceeding 3,500 m of altitude are characterised by a mountain climate with frequent frost and occasional snow (Hurni 1988), with mean daily temperature ranging from 1.5°C in the coldest day up to 14.6°C in the hottest day (Hurni and Stähli 1982). Rainfalls chiefly occur during the wet season (May-August), followed by a dry season, with mean annual values of 1,515 mm at Gich Camp (Hurni and Stähli 1982). Moving from the northern to the southern slopes of the Simien Mts., rainfalls are typically decreasing.

**Figure 1.**
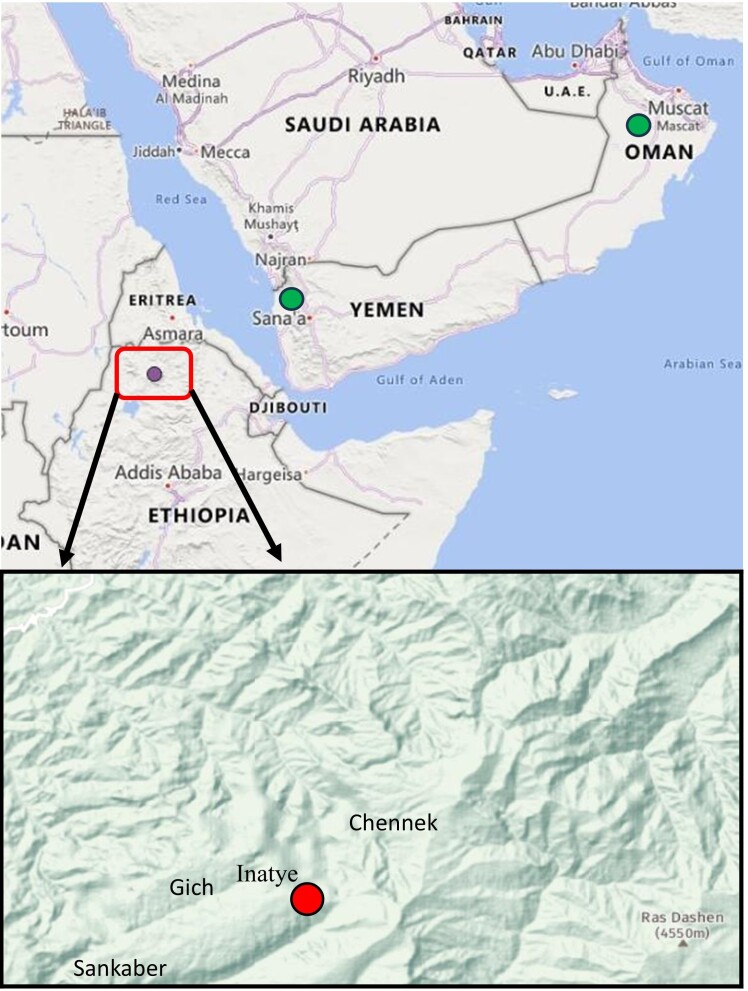
Overall range of *Diplotaxis kohlaanensis* according to literature and herbarium data (green dots) and field surveys (purple dot) and distribution map of *D. kohlaanensis* in Ethiopia (red dot).

Our field surveys were performed in December 2024 and January 2025. Flowering and fruiting specimens of *Diplotaxis kohlaanensis* were collected on the vertical cliffs below Mt. Inatye (Fig. 1). As it is located in rather inaccessible stands, it was decided to collect a limited quantity of specimens by harvesting only the elements relevant for the proper identification of the plant, such as basal and cauline leaves, flowers and siliques, also for avoiding any potential damage to the population. The collected specimens are preserved at the Herbarium of Catania (CAT, herbarium acronym follows Thiers (2025)).

The morphological investigations on the Ethiopian material belonging to *D. kohlaanensis* were carried out on five individuals. All the specimens were compared with data from the protologue (Miller and Nyberg 1994) and other relevant literature (Zohary 1966, Pottier-Alapetite 1979, Thulin 1993, Miller and Cope 1996, Pignatti et al. 2019, Brullo and Brullo 2020).

The comparison amongst the specimens belonging to *D. harra*, *D. crassifolia*, *D. lagascana* and *D. kohlaanensis* was carried out using both living material and dried specimens preserved in B, BR, CAT, E, K, LI, MA, PAL, SANT, SAV, US, W and WAG. In particular, the four species have been differentiated, based on the following characters: habitus, leaf and stem indumentum, leaf blade shape and size, leaf margins, corolla colour, petal size and silique size and shape (including beak length). In addition, the conservation status of *D. kohlaanensis* in Ethiopia was assessed by using the IUCN criteria (IUCN 2001) and the guidelines for regional application (IUCN 2003), according to the GeoCAT (Geospatial Conservation Assessment Tool) programme (Bachman et al. 2011). Finally, for the phytogeographic study, a checklist of the species occurring in the Afromontane and Afroalpine areas of the Simien Mts. exceeding 3,000 m a.s.l. was elaborated by using literature (Puff and Nemomissa 2005, Melese et al. 2018) and field surveys. Then, with the support of the POWO database, it was possible to identify species occurring in the Simien Mts. with a disjunct distribution range in eastern Africa and the Arabian Peninsula. Subsequently, for each species, the vegetation type was identified with the support of literature (Edwards and Hedberg 2009) and field data. Besides, the plant life form of these taxa has also been investigated. To compare the plant life forms of taxa with an East Africa and Arabian distribution, a stacked bar chart was generated using RStudio (RStudio Team 2020) and the tidyr and ggplot2 libraries (Wickham 2016, Wickham et al. 2017).

## Data resources

The following herbarium specimens have been examined: ***Diplotaxis harra* (Forssk.) Boiss.**: YEMEN: Gov. Hadhramout, Jol Plateau, on the pipeline rd. from Wadi Araf to Tawila fields, immediately S of the highest point and watershed, 15°09'53.8"N, 49°22'37.1"E, 3 September 2001, 1620 m alt., *N. Kilian, P. Hein & M. A. Hubaishan s.n.* (MA917618; WAG1970184!); OMAN: Dhofar, Mughsayl, volcanic escarpments and foothills, 16.891781 N 53.823051 E, 28 September 2015, *P. Escobar Garcia s.n.* (W201602241!); Dhofar, Shaat, Rass Saijr Cliffs, forest and clearings, 16.763547 N 53.602058 E, 28 September 2015, *P. Escobar Garcia s.n.* (W201602242!); Dhofar, Taquah, Way to Wadi Darbat, deciduous forest, 17.070599 N 54.446298 E, 27 September 2015, *P. Escobar Garcia s.n.* (W201602243!); Dhofar, Al Mughsayl, Wadi in fondo alla discesa, dopo Al Mughsayl, lungo le pendici e il letto di sinistra. Alt. 50-100 m, 10 September 2002, *M. Raffaelli, M. Tardeli, S. Mosti 851* (E00983905!); Dhofar, Wadi Mughsayl, alt. 0-250 m, 4 March 1994, *I. McLeish 3437* (E00121242); Dhofar, road from Taqa to Medinat al Haq, in rough grass at the road side, 500 m alt., 17,073 N 54,255 E, 7 October 1984, *R.A. Ash 185* (E00449433!); JORDAN: Elgi prope Petram, Wadi Musa, Arabia Petraea, 18 June 1909, *F. Nàbělek 1403* (SAV0004618!); TUNISIA: Sousse, ca. 7 km on the road Knais to Gabghoub, off-road to artificial lake. Open terrain with herbs and shrubs, along lake, 90 m alt., 35°40.3' N, 10°26.3' E, 2 April 2003, *J.J. Wieringa 4848* (BR0000025202021!); MOROCCO: Great Atlas, Gorges du Ziz between Rich and Er-Rachidia, surroundings of Tunnel du Leginaires, 21.1 km S Rich, 1280 m alt., 31°10' N, 04°23' W, 29 April 1993, *R. Vogt 10366 & C. Oberprieler 4814* (B100354760!); SOMALIA: Togdheer, Burao District, Gaan Libaah (Gacan Libaax), ca. 140 km from Hargyesa on Hargyesa-Buran, fields, 2000 m alt., 28 December 1977, *K. Elmi, M. Suliman 216* (WAG0338919!); ***Diplotaxis crassifolia* (Rafin.) DC.**: SICILY (ITALY): Sizilien: Bachschlucht hinter Porto Empedorle südwestl. Agrigento, rechte Talseite nahe dem Ausgang, 15 April 1965, 1620 m alt., *H.E. Metlesics s.n.* (LI253868!); Paternò sulle rupi calcaree oltre il Simeto, 1 May 1896, *F. Tornabene s.n.* (CAT3970!); Paternò, 15 Aprile 1894, *P. Baccarini s.n.* (CAT3971!); Pietraperzia, s.d., *s.c.* (PAL5624!); Torre di Gaffe, Licata (AG), 25 June 1991, *G. Certa, F. Gendusa, E. Pira s.n.* (PAL71359!); ***Diplotaxis lagascana* DC.**: SPAIN: Almería: Tabernas, Llanos del Duque, sobre substrato seco, pedregoso y salino, 20 March 1998, *C.Morales, C.Quesada, L.Baena, J.E.Linares s.n.* (SANT 40196!); Velez de Benaudalla (Espana, prov. Granada, Andalucia), alt. 50 m, campos de cultivo al lado de la carrettera, comunidade de terofitos, 17 March 1982, *A.M. Negrillo, P. Aroza, J. Hurtado 12186* (BR000027543092V!); Velez de Benaudalla (Espagne, prov. Granada, Andalucia), El Azud de Vèlez, 40 m alt., bords des chemins, sur des sols remuès riches en petites pierres, 28 January 1980, *M. Ladero, O. Socorro, J. Hurtado 10240* (BR000026282237!); Base de Sierra Grossa, Alicante, 11 November 1949, *S. Rivas s.n.* (US03584685!); ***Diplotaxis kohlaanensis* A.G. Mill. & J.A. Nyberg**: YEMEN: Amran to Kuhlan Road, limestone cliffs, 15 km E of Kuhlan, 2800 m alt., 26 March 1981, *A.G. Miller & D.G. Long 3213* (E0038048! holotype; K001291634! isotype); on cliffs above the town of Kohlaan, 2700 m alt., 31 January 1979, *J.R.I. Wood 2691*, identified by A. G. Miller (E00449402!); on a cliffs above Kohlaan, 2300 m alt., 17 February 1973, *J.R.I. Wood 2691*, identified by A. G. Miller (E00449405!); on cliffs on the S West edge of Jebel Mahdad, 3000 m alt., 26 September 1978, *J.R.I. Wood 2518*, identified by A. G. Miller (E00449403!); Bait Al Alama, J. Al Mahdad, 20 km W of Amran, SW facing sandstone cliffs, 2900 m alt., 26 September 1978, *A.G. Miller 236* (E00449404!); OMAN: Western Hajar Mountains, steep N-Exposed cliff in *Teucrio-Juniperetum*, 16 April 2011, *A. Patzelt s.n.* (E00702050!); ETHIOPIA: Simien Mt., rocky cliffs near the path between Inatye Mountain and Chennek, 3900 m alt., 13°15'20.17"N, 38°10'9.31"E, 23 December 2024, *S. Cambria s.n.* (CAT!).

## Taxon treatments

### Diplotaxis kohlaanensis

A.G. Miller & J.A. Nyberg 1994

D573DDCA-357C-511B-8B99-C6FFC5BB5F7C

#### Materials

- **Type status:** Other material. **Occurrence:** individualCount: 100; occurrenceID: 0DD55C97-CA41-5778-B3F7-F8CECAA94124; **Taxon:** scientificNameID: *Diplotaxis kohlaeensis*; kingdom: Plantae; order: Brassicales; family: Brassicaceae; genus: Diplotaxis; **Location:** continent: Africa; country: Ethiopia; locality: Simien Mountains; verbatimElevation: 3900 m; verbatimLatitude: 13°15'20.17"N; verbatimLongitude: 2 38°10'9.31"E; **Identification:** identifiedBy: S. Cambria; dateIdentified: 23-12-2024

#### Description

**Figure 2.**
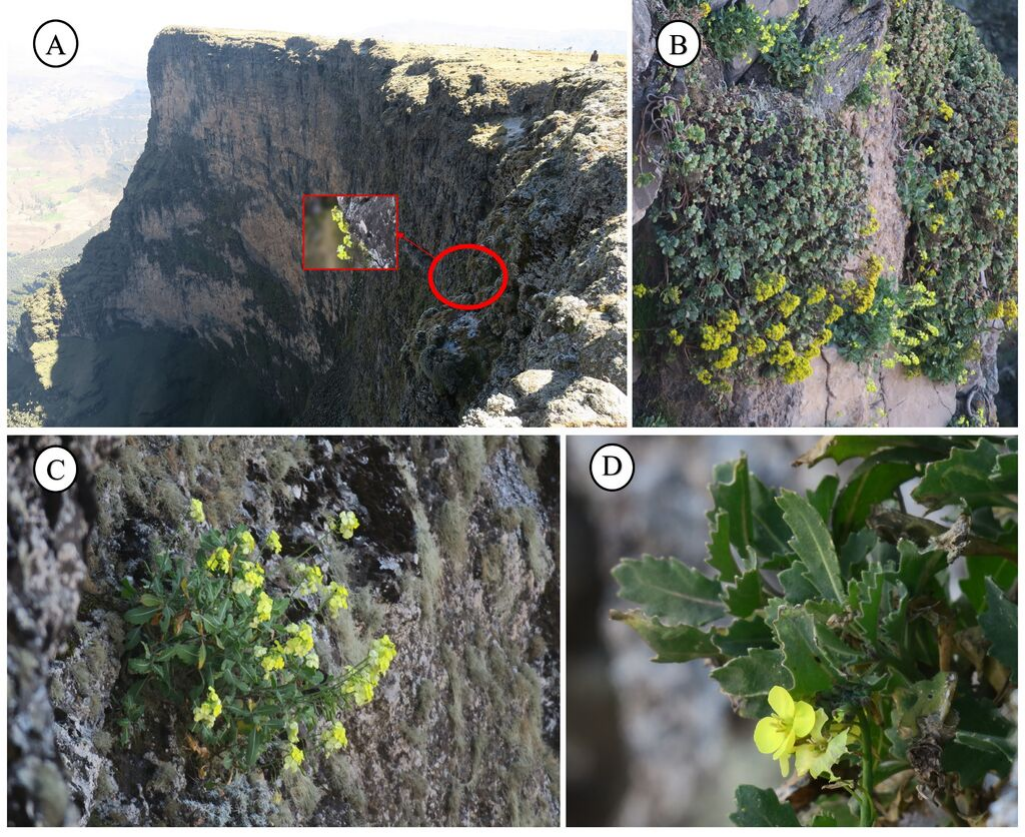
*Diplotaxis kohlaanensis* in the Ethiopian stands: **A** Natural habitat where an individual is highlighted by a red circle; **B** Chasmophilous vegetation with *Aeonium leucoblepharum* and *D. kohlaanensis*; **C** Habitus of *D. kohlaanensis*; **D** Detail of the leaves and flowers.

**Figure 3.**
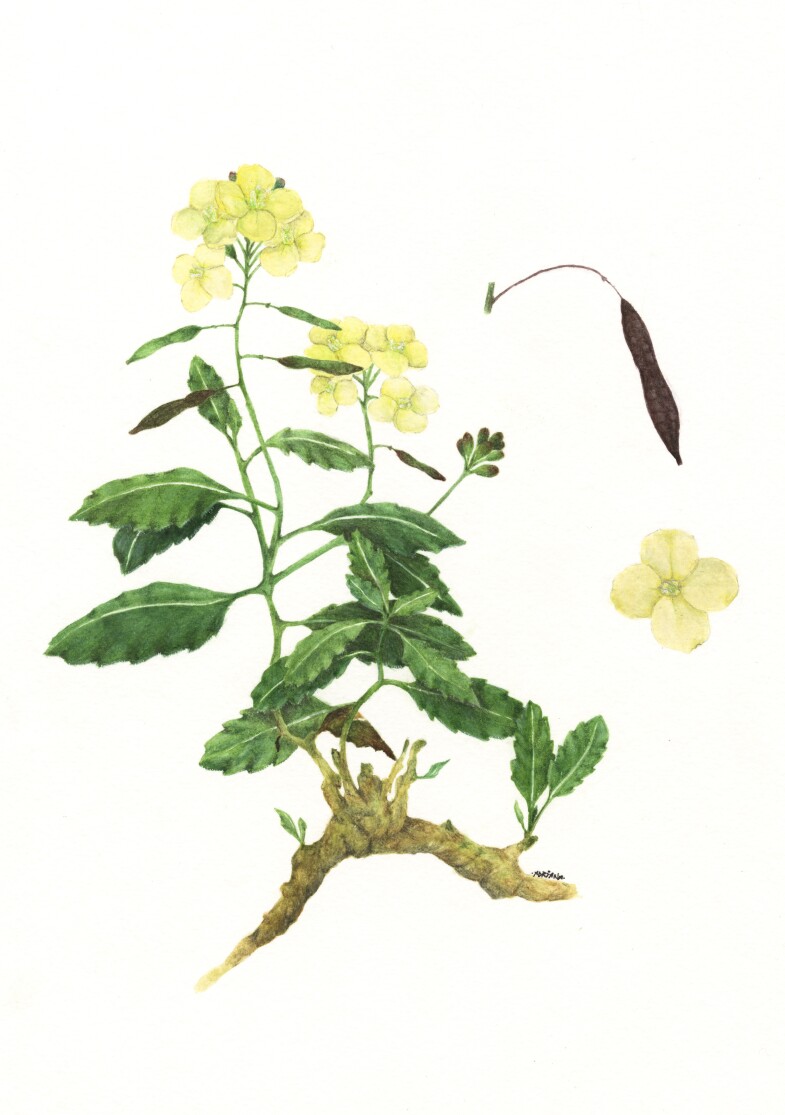
Iconography of *Diplotaxis kohlaanensis*, based on material from Ethiopia. Drawing by Adriana Santanello.

Shrubby and compact chamaephyte, generally glabrous, with many stems arising from a woody rootstock. Flowering branches erect or ascending, 30–60 cm long. Leaves slightly fleshy, glaucous, all petiolate with a well-marked whitish mid-rib. The lower ones with ovate to oblong-ovate blade, 15–80 × 10–20 cm, obtuse apex and serrate margin with 4–6 pairs of teeth or sinuate to entire, rarely with few scattered hairs. Upper leaves smaller, 5–40 × 5–10 mm, provided with a shorter petiole. Racemose inflorescence, erect or ascending, with the flowers overtopping the buds. Flower pedicels 10–14 mm long, equal or shorter than to the petals. Calyx with four sepals, 5–6 mm long, generally 2.5–3 times shorter than the petals, externally hairy particularly along the mid-rib and near the base. Inner sepals 1.8-2.5 mm broad and saccate at base, the outer 1.5–2 mm broad, with non-saccate base and clearly hooded tip. Corolla with four petals, pale yellow, broadly obovate and narrowing below into a linear claw, 10–14 × 5-6 mm, with rounded tip. Androecium consisting of 6 stamens; the median 8–11 mm long, with prominent nectarial glands at the base; the lateral 6–7 mm; anthers 2–3 mm long. Gynoecium with cylindrical ovary, glabrous, shortly stipitate; stigma bilobed. Siliques erect or patent, linear to linear-oblong, flattened, 12–40 × 2.5–3.2 mm, with the seeds in two rows; beak seedless, 1.5–2 mm long; Seeds pale reddish to brown, flattened, 1–1.5 × 0.5–l mm (Fig. 2, Fig. 3).

#### Distribution

Based on the current knowledge, the species is localised in Ethiopia on the vertical, basaltic, north-facing cliffs of the Simien Mts. at an altitude ranging from 3,700 to 3,900 m a.s.l.

#### Ecology

The species was found on the escarpment below Mt. Inatye, which is characterised by chasmophytic vegetation rich in rare and phytogeographically relevant species, such as *Aeonium leucoblepharum* Webb ex A.Rich., Arabis alpina L. subsp. alpina, *Campanula edulis* Forssk , *Poa simensis* Hochst. ex A.Rich., Helichrysum citrispinum Delile var. citrispinum, *Dianthus longiglumis* Delile, *Rosularia semiensis* (J.Gay ex A.Rich.) H.Ohba, *Asplenium aethiopicum* (Burm.f.) Becherer and marginally also Primula verticillata Hochst. subsp. simensis (Hochst.) W.W.Sm. & Forrest [=*Evotrochis simensis* (Hochst.) Fırat & Lidén].

#### Conservation

The population of *D. kohlaanensis* entirely falls within the Simien National Park, a protected area created in 1969, a period when 80% of the Park was subjected to human exploitation through livestock grazing, cultivation and settlements (Debonnet et al. 2006). Actually, this is still threatened by human activities, particularly grazing and logging (Jacob et al. 2016), further aggravated by the recent war events which led to a severe reduction of the wooded areas due to coppicing and fires, as highlighted by Meaza et al. (2025) for other mountains of northern Ethiopia. For this reason, the Simien Mountain National Park has been listed as a World Heritage Site in Danger since 1997. Despite this, the vertical cliffs are poorly affected by human disturbance due to their inaccessibility and, therefore, they represent the best-preserved environments of this area. The population of *D. kohlaanensis* contains a very low number of individuals (roughly estimated in ca. 100 mature plants) that, even if not immediately threatened, at this stage, we do not know how the species would respond to global warning. By using the Geospatial Conservation Assessment Tool (Bachman et al. 2011), the extent of occurrence (EOO) is calculated to be 0.028 km^2^, while the area of occupancy (AOO) is 4 km^2^. Following the IUCN Criteria at global and regional level (IUCN 2001, IUCN 2003), with the estimated EOO less than 100 km^2^, an AOO less than 10 km^2^, the conservation status of *D. kohlaanensis* for Ethiopia has been classified as Endangered at regional level (EN reg: D) according to criterion D.

#### Biology

Flowering and fruiting during the dry season from November to March.

## Identification Keys

### Key to the taxa belonging to *Diplotaxis harra* species complex

**Table:** 

1	Annual or rarely perennial herb, loosely branched, with densely hairy leaves and stems, leaves not fleshy with 5–15 pairs of teeth	*Diplotaxis harra*
–	Perennial suffruticose plant, with a basal woody rootstock, densely branched, with glabrous or glabrescent leaves and stems, leaves fleshy with 1–6 pairs of teeth or pinnatipartite	2
2	Flowering branches flexuosus, leaves with dentate or lobed margins, petals 7–9 mm long, shorter than flower pedicel, dark yellow, siliques hanging at fruiting, flowers not overtopping the buds	3
–	Flowering branches erect or ascending, leaves entire or with dentate margins and never lobed, petals 10–14 mm long, equal or longer than flower pedicel, pale yellow, siliques patent at fruiting, flowers overtopping the buds	*Diplotaxis kohlaanensis*
3	Lower leaves generally dentate, sometimes slightly lobed, upper leaves entire, linear and subsessile, siliques up to 6 cm long	*Diplotaxis crassifolia*
–	Lower leaves pinnatipartite to pinnatifidous, upper leaves markedly lobed and sessile or petiolate, siliques up to 4 cm long	*Diplotaxis lagascana*

## Analysis

The flora of the Simien Mts. includes more than 500 species (Puff and Nemomissa 2005, Masresha 2022), representing one of the most biodiverse area of the Ethiopian highlands, contributed by the remarkable variety of habitats ranging from the Afromontane forests, *Erica*-dominated scrublands, dry grasslands, Afroalpine vegetation, cliffs and wetlands.

This habitat heterogeneity is chiefly due to the peculiar climatic and topographic conditions that contribute to the genesis and isolation of several endemic species. In particular, the surveyed area hosts 75 endemic species of Ethiopia representing 14% of the total Siemen flora. The floristic value of the area is further underlined by the occurrence of 12 narrow endemic species, exclusively found in this area (Masresha 2022). As highlighted by Puff and Nemomissa (2001), Simien Mts. show a particular phytogeographic interest for the occurrence of many species, especially Afroalpine, also present in other eastern Africa mountains, as well as for taxa shared with the mountains of southern Africa (Drakensberg). Whereas at low-altitude stands, the Sudano-Zambezian floristic element is also well represented. Finally, an interesting group is represented by the so-called amphi-Red Sea species, i.e. species occurring on both eastern Africa and Arabian Peninsula (mainly the western escarpment of North Yemen), to which *Diplotaxis kohlaanensis* belongs. The “Arabian connection”, as highlighted by Wickens (1975), is so relevant from a phytogeographic viewpoint that it was used to gather the lowlands of these two territories in the same phytogeographical region, as confirmed by White (1978) who considered the mountains of western Yemen as part of the Ethiopian mountain system. This close biogeographical connection is explained by the complex geological history of this area. Actually, a rifting phase of the Arabian plate started at ~ 30 Ma (early Oligocene), with respect to Africa, thus giving origin to the Red Sea which therefore geologically represents a young basin, the first pulse of seafloor spreading occurring during Pliocene ∼ 4.6 Ma, around ∼ 17.1°N (current coordinates) and propagating southwards, separating the Danakil microplate from Arabia (Schettino et al. 2016, Schettino et al. 2019). Therefore, before the formation of the Red Sea, the Arabian Peninsula and eastern Africa formed a single landmass, as testified by the occurrence of numerous species belonging to an ancient Tertiary tropical flora, such as *Mimusops laurifolia* (Forssk.) Friis and *Dracaena ombet* Kotschy & Peyr. (Friis 1980).

Some floristic disjunctions between the NE Africa-south Arabia and Macaronesian Islands have been widely documented, likely demonstrating the past existence of a continuous subtropical vegetation belt along the southern Tethys, which was broken before the Pleistocene (Engler 1879, Hedberg 1961, Bramwell 1985, Thulin 1994, Demissew et al. 2006, Thiv et al. 2010). In fact, during this last period, East Africa and southern Arabia experienced a significant cooling, leading to an impoverishment of the subtropical flora and, at the same time, to the arrival of many species from the Mediterranean area (Axelrod 1975, Kürschner 1998, Hegazy and Lovett-Doust 2016, Ghazanfar 2024). Besides, the formation of land bridges between the two shores of the Red Sea due to the lowering of the sea level during the glaciations (Bar-Yosef 1987, Petraglia et al. 2010, Armitage et al. 2011) could have favoured the migration of numerous species between the two territories.

In order to quantify and characterise the Simien species having an eastern Africa-Arabian distribution, we performed in-depth research of literature data, as well field investigations (Table 1).

The Afromontane (3,000-4,000 m a.s.l.) and Afroalpine (> 4,000 m a.s.l.) areas of the Simien Mts. account for 300 taxa. Forty-seven species (ca. 15%) have a range extending from eastern Africa to the Arabian Peninsula, including some taxa with a punctiform extension to other areas of Africa and Madagascar. Within this contingent, three species are exclusively found in humid environments, six grow in the Afroalpine grasslands above the tree line, six in the scrublands (12%), 11 in forests and woodlands (23%), 13 in vertical cliffs (28%) and 16 are linked to the Afromontane grasslands (34%). The life-form analysis shows the prevalence of hemicryptophytes (38%), followed by phanerophytes (27%), chamaephytes (22%), therophytes (8%) and geophytes (4%).

**Figure 4.**
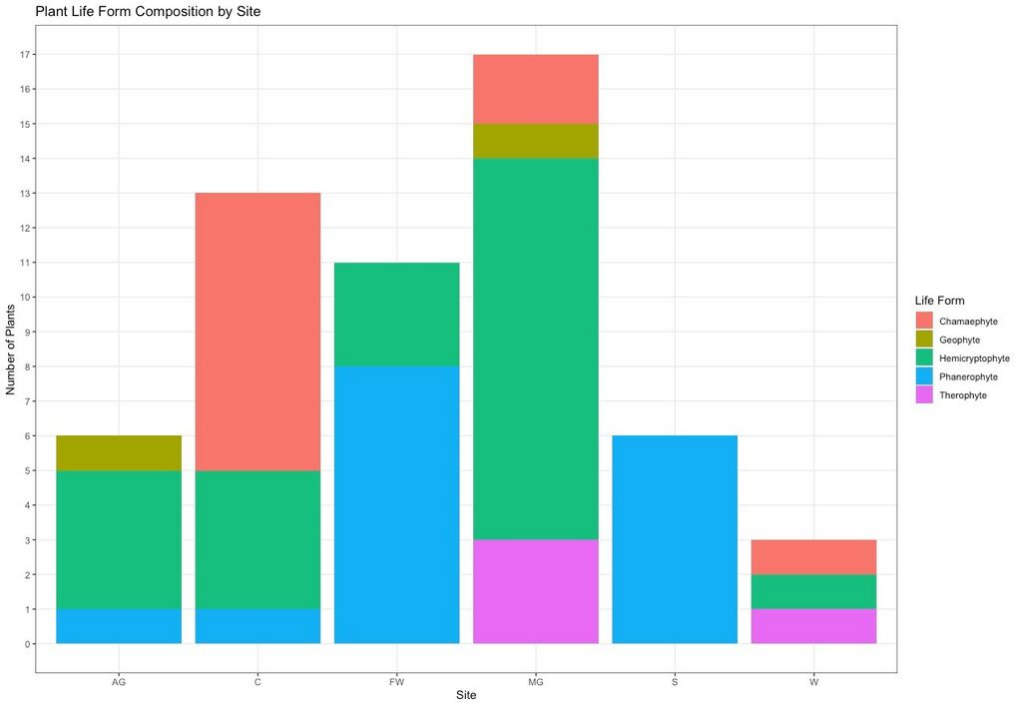
Eastern African-Arabian taxa in different habitats by life form. FW: Forests and woodlands; “AG”: Afroalpine grasslands; “MG”: Afromontane grasslands; “C”: cliffs; “WE”: Wet environments; “S”: Scrublands.

As concerns the exclusive E African-Arabian element, 31 taxa were detected. In particular, one (*Crassula granvikii* Mildbr.) is linked to humid environments and, in particular, to mountain streams (3%), four in scrublands (12%), five are present in Afroalpine meadows above the tree line (16%), six in forests and woods (19%), 10 are linked to Afromontane meadows (32%) and, lastly, 11 occur on vertical cliffs (36%). As regards the life-form, hemicryptophytes (39%), phanerophytes (25%) and chamaephytes (22%) prevail, while therophytes and geophytes are poorly represented. As expected, hemicryptophytes prevail in the Afromontane and Afroalpine grasslands, while phanerophytes dominate in woodlands and shrublands. As concerns the cliffs, a clear prevalence of chamaephytes was detected (Fig. 4). Finally, only five species are exclusive to Ethiopia and the southern Arabian Peninsula (particularly N Yemen): *Clematis longicauda* Steud. ex A.Rich, *Dianthus longiglumis*, *Diplotaxis kohlaanensis*, *Disa pulchella* Hochst. ex A.Rich. and *Tenaxia subulata* (A.Rich.) N.P. Barker & H.P. Linder.

## Discussion

**Figure 5.**
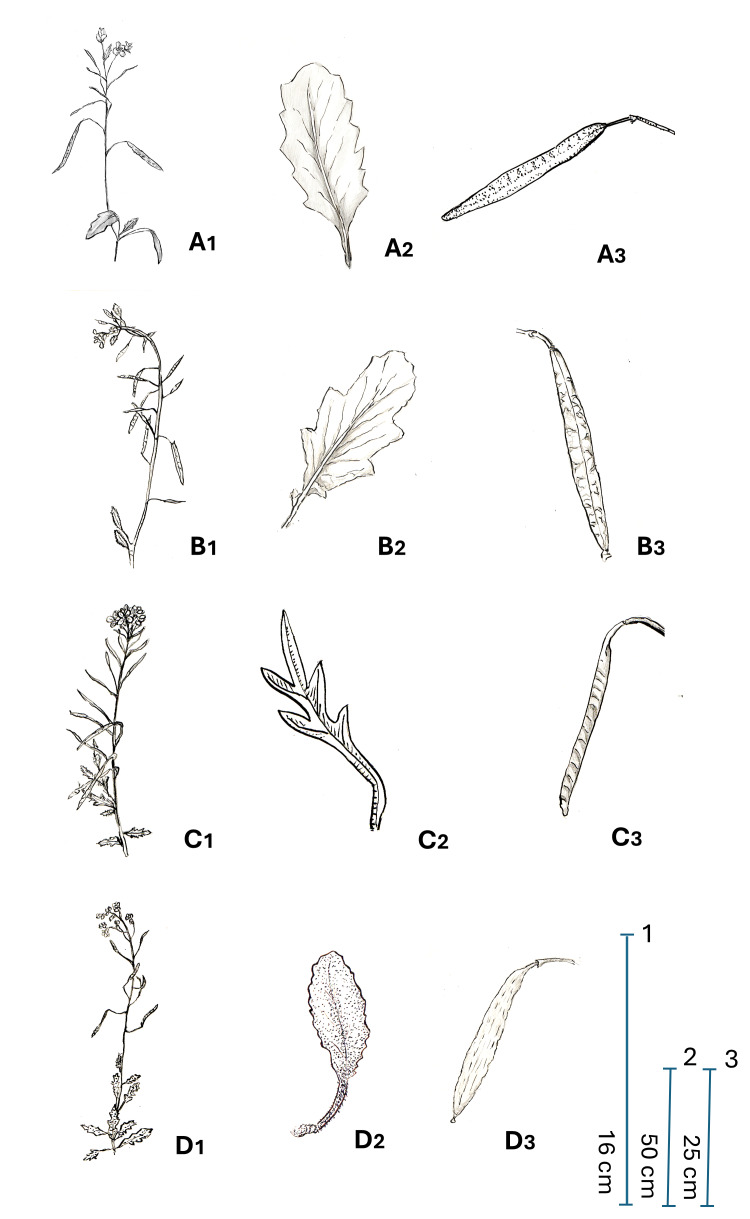
Main morphological features of the taxa belonging to the *Diplotaxis harra* group - flowering branches (1); Leaf (2); Silique (3): **A** *D. kohlaanensis*; **B** *D. crassifolia*; **C** *D. lagascana*; **D** *D. harra*. Drawings by Rosaria Di Cicca.

The plants found and sampled in Ethiopia correspond well with *Diplotaxis kohlaanensis* described by Miller and Nyberg (1994). In particular, the most relevant features of this species are the suffruticose habit, generally glabrous and dentate leaves with 4–6 teeth per side, erect and slightly flexible flowering branches, flowers with pale yellow petals, longer than 10 mm, as well as the size of the siliques. However, the Ethiopian plants sometimes present a sparse hairiness on the margin and the lower side of the leaves; furthermore, the stems do not reach the length described by the above-mentioned authors (up to 3 m), reaching a maximum of 50–60 cm, being likely adapted to the more severe climatic conditions. In general, according to our analyses, the species is very distinct from the other taxa belonging to the *D. harra* group (Table 2, Fig. 5), especially for the flower features, i.e. petals longer than 10 mm, flowers overtopping the buds and floral peduncles equal to or shorter than the petals. Conversely, other features, such as silique size, beak length and hairless leaves and stems, seem to be taxonomically meaningless. In fact, the silique of *D. kohlaanensis* is 1.2–4 cm long, while the beak can reach 2 cm, similarly to *D. crassifolia* and *D. lagascana*. Furthermore, even quite rarely, the species can present a sparse hairiness on the leaves margins, as well as other taxa of the *D. harra* complex being hairless, making this character not taxonomically relevant. However, *D. kohlaanensis* is very easily distinguished from *D. harra*, although this is a really polymorphic species whose morphological variability of the leaves has been already highlighted by Oberprieler et al. (2017). In addition, *D. harra* is an annual or biennal species, densely hairy and hispid, with leaves mostly basal, coarsely dentate with a high number of teeth (5–15 pairs). Even from the ecological viewpoint, *D. harra* appears to be rather distinct, being linked to sandy or rocky habitats in very dry stands, while D. *kohlaanensis* exclusively grows in the high mountain cliffs featured by a cool and humid climate. As regards the population of *D. harra* reported in Somalia by Thulin (1993), the record seems rather doubtful, since the herbarium material (WAG0338919!) and the description provided by the author did not match with *D. harra*. Therefore, for a proper and reliable identification of the Somali population, further studies are required.

The two European species of the *D. harra* complex, namely *D. crassifolia* and *D. lagascana*, show a greater morphological affinity with *D. kohlaanensis*, especially for the subshrubby habit and the glabrous (or glabrescent) and fleshy leaves. *D. lagascana*, a Spanish endemism often treated at subspecific rank (sub D. harra (Forssk.) Boiss. subsp. lagascana (DC.) O.Bolòs & Vigo) is easily distinguishable for having pinnatipartite to pinnatifidous leaves (Castroviejo et al. 1993). As concerns *D. crassifolia* [=D. harra (Forssk.) Boiss. subsp. crassifolia (Raf.) Maire], a Sicilian endemism (Pignatti et al. 2019, Brullo and Brullo 2020), wrongly recorded also for North Africa (Maire 1965, Pottier-Alapetite 1979, Fennane 1999), shows a similar leaf blade, often dentate with 1–6 teeth for side. However, it is easily distinguished from *D. kohlaanensis* by its long, flexible and pendulous flowering branches and by its flowers with dark yellow and much shorter petals (6-10 mm), always shorter than the peduncles. In conclusion, the species at issue is very well distinguishable from the other taxa of the *D. harra* group, not only for its morphology, but also for its ecological behaviour and geographical distribution. Therefore, the discovery of this specie in Ethiopia has a remarkable phytogeographic value, since it is the first record of a *Diplotaxis* species in Ethiopia and, likely most interesting, confirms the phytogeographic relationships between the Ethiopian highlands and the Arabian Peninsula mountains.

Our investigations on the Afromontane and Afroalpine species with an East African-Arabian distribution (i.e. amphi-Red Sea taxa) occurring in the Simien Mts. highlight the clear prevalence of perennial, herbaceous or woody species, mainly linked to the Afromontane grasslands and woodlands. This is due to the lack of mountains in the Arabian Peninsula higher than 3,600 m a.s.l., not allowing the presence of a significant Afroalpine flora typical of the highest peaks of the Simien Mts.. Therefore, the mountain flora in common is mostly represented in the altitudinal belt covered by *Erica* woods, shrubs and secondary grasslands. Conversely, the significant occurrence of chamaephytes must be highlighted showing this distribution in vertical cliffs even at altitudes typical of the Afroalpine belt. In fact, this environment represents a well-known conservative and stable habitat (Larson et al. 2000, García et al. 2020, Sangüesa‐Barreda et al. 2022, Múgica Carnicero et al. 2024), where, due to the minor human disturbance and the peculiar microclimate, ancient species, often endemic, have been preserved, providing precious evidence of a flora which have now largely disappeared in the nearby areas. In this perspective, *Diplotaxis kohlaanensis* can be interpreted as a relict species, linked to particularly cool and humid microclimate typical of high-altitude north-exposed cliffs. Furthermore, it shows some archaic features within the *Diplotaxis* genus, such as the woody habit (Lems 1960, Bramwell 1972, Cronk 2006, Cronk 2008, Lens et al. 2013), suggesting a potential ancestry compared to some allied Mediterranean herbaceous species, as already highlighted by Pignatti (1979) and by Bramwell (1976) for some Macaronesian-African taxaBramwell (1976). Actually, the phytogeographical affinities between the Mediterranean area and the Horn of Africa have already been highlighted by several authors (Balfour 1888, Gillett 1941, Lavranos 1975, Fici 1991), particularly for the northern Somali escarpment, an area with several taxa having close Mediterranean allies. According to Wickens (1975), the southern Arabian Peninsula and eastern Africa represented an important centre of refuge, speciation and radiation of Holarctic taxa during the glacial phases of Quaternary. Even today, these species strongly characterise the flora of the Ethiopian plateau, which, compared to other mountain ranges of eastern Africa (Hedberg 1957, Hedberg 1964, Hedberg 1969, Bussmann 2006, Cambria et al. 2024) shows a lower incidence of the typical Afroalpine flora and a more significant presence of the Holarctic element. In this frame, the discovery of *D. kohlaanensis* suggests the role of the high altitude cliffs of the Siemen Mts. as a refuge habitat for several species with northern affinities, whose origin probably date back to the glacial phases of Pleistocene when, as a result of a relatively cool and humid climate, numerous species migrated from the north through the Red Sea corridor reaching the Arabian Peninsula and the Horn of Africa. This origin can be postulated also for other species growing in the same rupicolous habitat of *D. kohlaanensis*, such as *Dianthus longiglumis* Delile, *Primula verticillate* Forssk., *Stachys richardiana* R.Kr. Singh, *Sonchus melanolepis* Fresen, *Arabis alpina* L. etc. As concerns *Aeonium leucoblepharum* Webb ex A.Rich., frequent in the same environments, its marked geographical isolation from all other species of its genus should be considered as the effect of a long-distance dispersal during the Pleistocene, rather than as the result of older ancient phytogeographic connections between the Macaronesian Islands and East Africa (Mort et al. 2002). Further research in the Siemen Mts. escarpment may lead to other important floristic findings, also complemented by the most recent technological tools such as drones, which have proven particularly useful in the investigation of similar inaccessible vertical cliffs (Porrovecchio et al. 2024, Tavilla et al. 2024, Wagner et al. 2024). Likewise, phylogenetic studies aiming at clarifying the relationships between *D. kohlaanensis* and the other taxa of *D. harra* complex are highly desirable.

## Supplementary Material

XML Treatment for Diplotaxis kohlaanensis

## Figures and Tables

**Table 1.** List of species with a disjunct range in East Africa and the Arabian Peninsula present in the highlands of the Simien Mountains. Plant communities are abbreviated as follows: FW: Forests and woodlands; “AG”: Afroalpine grasslands; “MG”: Afromontane grasslands; “C”: cliffs; “WE”: Wet environments; “S”: Scrublands.

**Taxa**	**Plant communities**	**Life-form**	**Notes**
*Aeonium leucoblepharum* Webb ex A.Rich.	C	Chamaephyte	
*Alchemilla cryptantha* Steud. ex A.Rich	W, MG	Chamaephyte	Also in other mountain area of central and southern Africa
*Anthospermum pachyrrhizum* Hiern	C	Chamaephyte	
*Artemisia abyssinica* Sch.Bip. ex Oliv. & Hiern	MG	Therophyte	
*Astragalus atropilosulus* (Hochst.) Bunge	MG	Hemicryptophyte	Also in south-eastern Africa
*Buddleja polystachya* Fresen.	S	Phanerophyte	
*Campanula edulis* Forssk.	C	Hemicryptophyte	
*Cerastium octandrum* Hochst. ex A.Rich.	MG	Therophyte	Also in Central Africa
*Chrysopogon plumulosus* Hochst.	MG	Hemicryptophyte	Also in Central Africa
*Clematis longicauda* Steud. ex A.Rich	C, FW, S	Phanerophyte	
*Clematis simensis* Fresen.	FW, S	Phanerophyte	It occurs also in Central Africa
*Clinopodium abyssinicum* (Benth.) Kuntze	MG	Hemicryptophyte	
*Clutia lanceolata* Forssk.	FW	Phanerophyte	
*Colchicum schimperianum* (Hochst.) C.Archer	AG	Geophyte	
*Crassula alba* Forssk.	C	Hemicryptophyte	Also in southern Africa
*Crassula granvikii* Mildbr.	W	Therophyte	
*Crepis rueppellii* Sch.Bip.	MG	Hemicryptophyte	
*Daucus melananthus* (Hochst.) Reduron, Spalik & Banasiak	MG	Hemicryptophyte	Also in Madagascar, southern and western Africa
*Dianthus longiglumis* Delile	C	Chamaephyte	
*Diplotaxis kohlaanensis* A.G.Mill. & J.A.Nyberg	C	Chamaephyte	
*Disa pulchella* Hochst. ex A.Rich.	FW	Hemicryptophyte	
*Euphorbia petitiana* A.Rich.	MG, AG	Hemicryptophyte	
*Euphorbia schimperiana* Scheele	MG	Therophyte	Also in western Africa
*Geranium arabicum* Forssk.	FW	Hemicryptophyte	Also in Madagascar and western Africa
*Gladiolus abyssinicus* (Brongn. ex Lem.) B.D.Jacks.	MG	Geophyte	
*Gymnosporia arbutifolia* (Hochst. ex A.Rich.) Loes.	FW	Phanerophyte	
*Helichrysum forskaohlii* (J.F.Gmel.) Hilliard & B.L.Burtt	FW	Phanerophyte	Also in central and western Africa
*Helichrysum schimperi* (Sch.Bip. ex A.Rich.) Moeser	FW	Phanerophyte	
*Helichrysum splendidum* Less.	AG	Phanerophyte	Also in southern Africa
*Hypericum revolutum* Vahl	FW	Phanerophyte	It occurs also in the western part of Africa
*Juniperus procera* Hochst. ex Endl.	FW	Phanerophyte	It also extends into the south-eastern part of Africa
*Justicia ladanoides* Lam.	MG	Chamaephyte	Also in central and western Africa
*Minuartia filifolia* (Forssk.) Mattf.	C	Chamaephyte	
*Polypogon schimperianus* (Hochst. ex Steud.) Cope	W	Hemicryptophyte	Also in south-eastern Africa
*Evotrochis verticillata* Forssk.	C	Hemicryptophyte	The Ethiopian population are sometimes considered a different species named *E. simensis* (Hochst.) Fırat & Lidén
*Rosa abyssinica* R.Br. ex Lindl.	S	Phanerophyte	
*Rumex nervosus* Vahl	S	Phanerophyte	
*Salvia merjamie* Forssk.	AG, MG	Hemicryptophyte	
*Salvia schimperi* Benth.	MG, C	Hemicryptophyte	
*Senecio schimperi* Sch.Bip. ex A.Rich.	AG	Hemicryptophyte	
*Silene flammulifolia* Steud. ex A.Rich.	C	Chamaephyte	
*Silene macrosolen* Steud. ex A.Rich.	C	Chamaephyte	
*Sonchus melanolepis* Fresen.	C	Chamaephyte	
*Tenaxia subulata* (A.Rich.) N.P.Barker & H.P.Linder	AG, MG	Hemicryptophyte	
*Thesium radicans* Hochst. ex A.Rich.	MG	Hemicryptophyte	
*Trifolium semipilosum* Fresen.	FW, MG	Hemicryptophyte	
*Vernonia bipontini* Vatke	S	Phanerophyte	

**Table 2.** Main diacritical characters of taxa belonging to *Diplotaxis harra* complex.

	***D. harra***	***D. lagascana***	***D. crassifolia***	***D. kohlaanensis***
**Habitus**	annual or perennial	suffruticose	suffruticose	suffruticose
**Leaf margin and shape**	dentate with 5–15 pairs of teeth	pinnatipartite to pinnatifidous	dentate with 1–6 pairs of teeth or pinnatipartite	dentate with 4–6 pairs of teeth
**Leaf indumentum**	densely hairy	glabrous or glabrescent	glabrous or glabrescent	glabrous or glabrescent
**Lower leaf size (mm)**	10–120 × 5–50	25–110 × 10–25	10–60 × 5–15	15–80 × 10–20
**Flowering branches length (cm)**	60	80	100	300
**Inflorescence**	erect	erect	flexuous and hanging	erect
**Petal colour**	dark yellow	dark yellow	dark yellow	pale yellow
**Petal length (mm)**	7–9	7–9	6–10	10–14
**Siliques size (mm)**	10–50 × 2–3	25-40 × 1.5–3.5	30–60 × 2–3	12–40 × 2.5–3.2
**Beak length (mm)**	1.5–2	1–2	1–2	1.5–2
